# Transcriptome Profiling of a Salt Excluder Hybrid Grapevine Rootstock ‘Ruggeri’ throughout Salinity

**DOI:** 10.3390/plants13060837

**Published:** 2024-03-14

**Authors:** Pranavkumar Gajjar, Ahmed Ismail, Tabibul Islam, Md Moniruzzaman, Ahmed G. Darwish, Ahmed S. Dawood, Ahmed G. Mohamed, Amr M. Haikal, Abdelkareem M. El-Saady, Ashraf El-Kereamy, Sherif M. Sherif, Michael D. Abazinge, Devaiah Kambiranda, Islam El-Sharkawy

**Affiliations:** 1 Center for Viticulture and Small Fruit Research, College of Agriculture and Food Sciences, Florida A&M University, Tallahassee, FL 32308, USA; pranavkumar1.gajjar@famu.edu (P.G.); ahmed.ismail@ucr.edu (A.I.); md.moniruzzaman@famu.edu (M.M.); ahmed.darwish@famu.edu (A.G.D.); ahmed.mohamed2@famu.edu (A.G.M.); 2 Department of Botany and Plant Sciences, University of California Riverside, Riverside, CA 92521, USA; ashrafe@ucr.edu; 3 Department of Horticulture, Faculty of Agriculture, Damanhour University, Damanhour 22516, Egypt; amr.haikal@agr.dmu.edu.eg; 4 Plant Sciences Department, University of Tennessee, Knoxville, TN 37996, USA; islamt@utk.edu; 5 Department of Biochemistry, Faculty of Agriculture, Minia University, Minia 61519, Egypt; 6 Horticulture Department, Faculty of Agriculture, Al-Azhar University, Cairo 11884, Egypt; ahmed8dawood@azhar.edu.eg; 7 Fertilization Technology Department, National Research Center (NRC), Cairo 12622, Egypt; elsaadyam@nrc.sci.eg; 8 Alson H. Smith Jr. Agricultural Research and Extension Center, School of Plant and Environmental Sciences, Virginia Tech, Winchester, VA 22602, USA; ssherif@vt.edu; 9 School of the Environment, Florida A&M University, Tallahassee, FL 32307, USA; michael.abazinge@famu.edu; 10 Department of Plant and Soil Sciences, Southern University Agricultural Research and Extension Center, Baton Rouge, LA 70813, USA; devaiah_kambiranda@subr.edu

**Keywords:** grapevine rootstock, Ruggeri, photosynthesis, carbohydrates, ROS detoxification, salinity, transcriptome profiling

## Abstract

Salinity is one of the substantial threats to plant productivity and could be escorted by other stresses such as heat and drought. It impairs critical biological processes, such as photosynthesis, energy, and water/nutrient acquisition, ultimately leading to cell death when stress intensity becomes uncured. Therefore, plants deploy several proper processes to overcome such hostile circumstances. Grapevine is one of the most important crops worldwide that is relatively salt-tolerant and preferentially cultivated in hot and semi-arid areas. One of the most applicable strategies for sustainable viticulture is using salt-tolerant rootstock such as Ruggeri (RUG). The rootstock showed efficient capacity of photosynthesis, ROS detoxification, and carbohydrate accumulation under salinity. The current study utilized the transcriptome profiling approach to identify the molecular events of RUG throughout a regime of salt stress followed by a recovery procedure. The data showed progressive changes in the transcriptome profiling throughout salinity, underpinning the involvement of a large number of genes in transcriptional reprogramming during stress. Our results established a considerable enrichment of the biological process GO-terms related to salinity adaptation, such as signaling, hormones, photosynthesis, carbohydrates, and ROS homeostasis. Among the battery of molecular/cellular responses launched upon salinity, ROS homeostasis plays the central role of salt adaptation.

## 1. Introduction

Salt stress negatively contributes to agriculture worldwide and, hence, represents one of the major threats to plant productivity. Salt accumulation in soil solution results in ionic anxiety (sodium–Na^+^ and chloride–Cl^−^), osmotic stress (physiological drought), and the interaction between both pressure types [1,2,3]. Further, salinity could be escorted by other stresses such as heat and/or drought. In addition, similar to other biotic/abiotic stresses, salinity leads to oxidative stress as the generation of excessive reactive oxygen species (ROS) [4,5]. Approximately 65–87% of the global crop production is annually lost due to plant stresses through which salinity only affects more than 833 million hectares worldwide, most notably in (semi)-arid regions [6,7]. It hinders plant growth and development by impairing critical biological processes such as photosynthesis, energy, and water/nutrient acquisition that ultimately culminate in cell death when stress intensity becomes uncured [8,9].

To survive under salinity, plants have to respond effectively by recruiting a set of proper actions [3,8]. The rapid access of NaCl into the root system triggers a battery of signal cascades that act in harmony with cellular secondary messengers such as Ca^2+^, H^+^, ROS, and plant hormones. Moreover, the rise of NaCl at the cellular level can act to a certain extent as a cheap osmolyte to counteract NaCl-imposed osmotic stress, hence turning a foe into a friend [2,10,11]. However, such a rapid influx of NaCl must be restricted to prevent its cellular accumulation, along with the fast decline in leaf stomatal conductance to restrict water loss [12]. Furthermore, NaCl must be effluxed out of the cell, compartmentalized into the vacuole, and damped inside old leaves [8]. Likewise, plants have evolved other efficient machinery to maintain redox homeostasis and osmotic balance. For instance, ROS detoxification is achieved by engaging antioxidant enzymes (e.g., superoxide dismutase-SOD, catalase-CAT, glutathione reductase-GR, glutathione peroxidase, and ascorbate peroxidase-APX) and non-enzymatic antioxidants, including stilbenes, flavonoids, carotenoids, and sugar alcohols [13,14]. Similarly, osmotic adjustment can be executed by accumulating osmolytes (osmoprotectants) such as proline, proline betaine, glycine betaine, alanine betaine, pipecolate betaine, hydroxyproline betaine, trehalose, and polyols [15].

Grapevine (*Vitis vinifera*) is one of the most important crops worldwide. The United States was the world’s third-largest grape cultivator in 2023, with roughly 6.9 million tons (www.worldpopulationreview.com; accessed on 5 March 2024). Grapes are relatively salt-tolerant, known for their higher sensitivity to Cl^−^ toxicity than Na^+^ [16]. The hot and semi-arid areas are the grapes’ preferentially cultivated regions, where soil salinization is predicted to be exaggerated due to global climate change [17]. Unfortunately, some 25–73% of current Mediterranean grapevine-growing regions are at risk of becoming uncultivable due to soil desertification, a climate-change-related process in which different types of water-related stresses (e.g., salinity and drought) can coexist [18]. Hence, their production is challenged by several types of plant stresses, most notably water-related stresses [19]. Several practical measures are taken to alleviate the negative impacts of stresses such as salt- and drought-related damages. For instance, economically important salt-sensitive cultivars are grafted on rootstocks derived from salt-tolerant sand grape (*V. rupestris*) and winter grape (*V. berlandieri*) [16,20]. In our previous study, we evaluated the physiological impacts of salinity on two grape hybrid rootstocks, 140 Ruggeri (*V. berlandieri* × *V. rupestris*) and Millardet et de Grasset 420A (*V. berlandieri* × *V. riparia*) [21]. They were considered highly competent salt excluder rootstocks that reduce salt accumulation in the leaves and berries of their grafted scions [22,23]. We showed that RUG maintains higher photosynthetic capacity under salinity and, accordingly, accumulates more sugars [21]. In the current study, we extend our efforts to understand the molecular events that occurred throughout a regime of salt stress followed by re-watering. Therefore, transcriptomic data was generated to address in time-resolved detail the response of grapevine rootstock RUG to salinity and recovery treatments. The pairwise comparisons between transcriptome profiles of salt-stressed on non-stressed RUG showed substantial changes in transcriptomic profiles, particularly after 24 h and 48 h. The employment of the WGCNA system biology approach linked the transcriptome data with the previously published results [21] (i.e., carotenoids, Fru, TSS, GPX, SOD, and Pro) demonstrated a considerable enrichment of Biological process GO terms (BP GOs) related to salinity adaptation in the positively correlated modules M1 and M11. For instance, the BP GOs of hormones (e.g., “hormone-mediated signaling pathway” and “ethylene-activated signaling pathway”), polysaccharide (e.g., “cell wall polysaccharide metabolic process”), photosynthesis (e.g., “chlorophyll metabolic process”, “chlorophyll biosynthetic process”, “carotenoid metabolic process”, “pigment metabolic process”, and “pigment biosynthetic process”), and ROS detoxification (e.g., “superoxide metabolic process”, “response to superoxide”, “removal of superoxide radicals”, “cellular response to superoxide”, “cellular response to oxygen radical”, and “response to reactive oxygen species”) were highly represented in both modules. The considerable enrichment of BP GO terms for signaling, hormones, photosynthesis, carbohydrates, ROS homeostasis, and other stress-related terms in M1 and M11 modules and their associated K-means clusters pointed to a battery of molecular/cellular responses established during salt stress. However, the ROS homeostasis activity stemmed from a wide range of gene-encoding proteins of different pathways, highlighting the central roles of ROS detoxification and homeostasis mechanisms in salt adaptation. This work would help in promoting the generation of new salt-adapted rootstocks to better cope with global climate changes.

## 2. Results

### 2.1. Global Changes in RUG Transcriptome during Salt Stress

In the current study, we investigated the changes in the transcriptome profile of RUG rootstock throughout salinity and recovery procedures to acquire an overview of the associated molecular events underlying its physiological responses. All collected samples were used for the RNA-seq experiment, excluding 8 h salt stress and 8 days recovery. Unless otherwise specified, the sampling order R1–R8 will refer to the sampling time; control, 0 h, 0.5 h, 2 h, 24 h, 48 h, 4 d, and 12 d, respectively.

**Figure 1:**
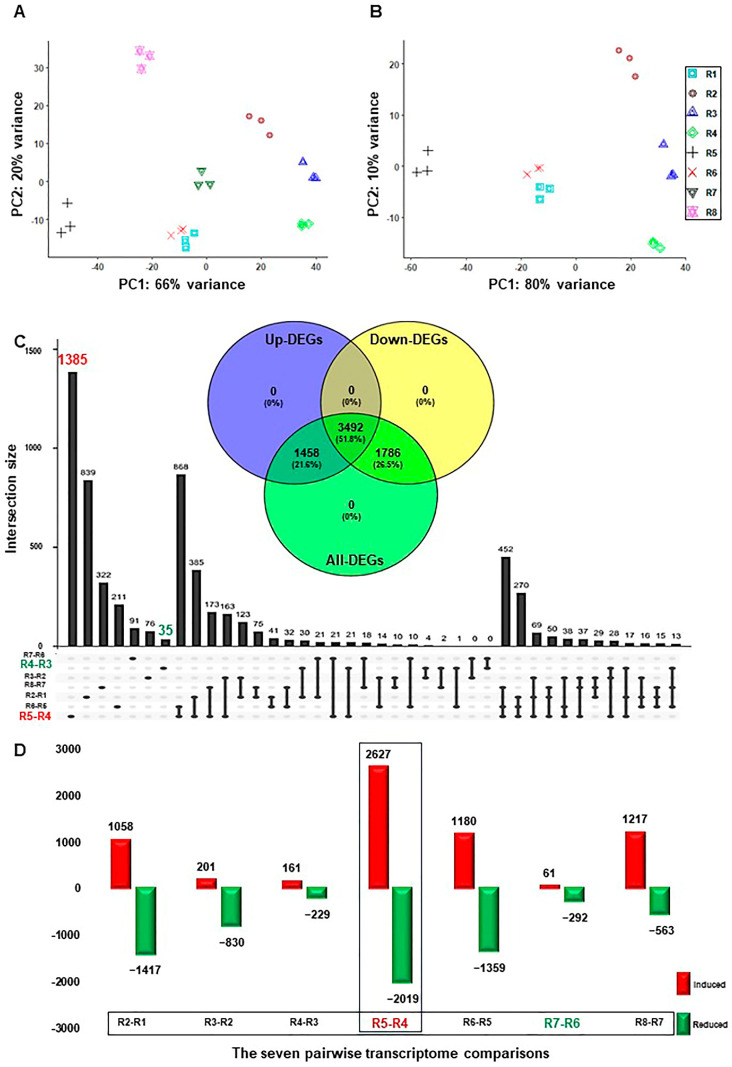
Temporal dynamics of RUG rootstock transcriptome during salt and recovery treatments. (**A**) Principal Component Analysis (PCA) showing the global similarity among RNA-seq samples of RUG rootstock, where salt stress was applied gradually, followed by a recovery procedure. (**B**) The PCA of the transcriptome data after excluding the recovery samples. (**C**) The Upset plot of the total number of the differentially expressed genes (DEGs) of each comparison. By using the DESeq2 pipeline, each two consecutive time points during the salinity and recovery procedure were compared, resulting in 13,214 (6736 non-redundant), with |log2FC| > ±1.5 and *p*-adjusted < 0.05. The included Venn diagram showed the non-redundant DEGs among the total, up-, and down-regulated transcripts. (**D**) Bar plots of DEGs that demonstrated the temporal expression patterns of genes in RUG during salinity and recovery procedures. The sampling order R1–R8 refers to the sampling time as follows: control, 0 h, 0.5 h, 2 h, 24 h, 48 h, 4 days, and 12 days, respectively. Each time point during the salinity and recovery treatments was given a distinctive color and symbol.

The sequenced, pair-ended 24 libraries (3 biological replicates × 8-time points) resulted in 620.1 Gb of high-quality clean data ranging from 21.1 Gb to 35.3 Gb per replicate. The clean reads were aligned to the V. vinifera reference genome sequence and projected to its transcriptome using STAR, which resulted in an 84.2% to 90.8% mapping rate (Table S1) [24,25]. Subsequently, the output transcriptomic coordinates files were quantified by Salmon in alignment mode [26]. Samples hierarchical clustering showed progressive changes in their transcript abundance during salt stress (Figure S1). Likewise, the principal component analysis (PCA) exhibited high consistency among transcript profiles that were separated along two main components. The first component (PC1) was accountable for 66% of the variance and was associated with the course of salinity (Figure 1A). The second component (PC2) was responsible for 20% of the variance and was essentially linked to the effect of recovery and sampling time. Interestingly, when the two recovery samples (R7 and R8) were excluded, the PC1 increased by 14% and, therefore, represented 80% of the variance (Figure 1B). Contrary, the PC2 diminished by half, signifying 10% of the variance. The data confirmed that salinity causes the primary source of variance with more than 65%, while the recovery treatment counteracts its impact by ~10–14%. However, other factors, such as sampling time and/or the circadian clock, impact the variance by 10% only.

Seven pairwise transcriptome comparisons between each consecutive time point of salinity and recovery (R2–R1, R3–R2, R4–R3, R5–R4, R6–R5, R7–R6, or R8–R7) resulted in 13,214 and 6736 redundant and non-redundant differentially expressed genes (DEGs) with PFDR < 0.05 and log2fold change > ±1.5, respectively (Table S2). The total non-redundant DEGs (6736) were approximately equal to the number of redundant up- (6505) and down-regulated (6709) transcripts. Interestingly, ~52% of the non-redundant DEGs (3492 out of 6736) showed up- or down-regulation at one of the studying points (Figure 1C). In addition, the total and exclusive number of DEGs, and the up- and down-regulated transcripts in each comparison differed significantly throughout stress (Figure 1C,D and Figures S2 and S3). For instance, the R5–R4 comparison showed the highest number of up- (2627) and down-regulated (2019) transcripts, which roughly represented one-third of the total number of redundant DEGs (Figure 1D). Similarly, the highest number of unique DEGs (1385), and the up- (1574) and down-regulated (1661) genes, respectively, was also detected in the R5-R4 comparison (Figure 1C and Figures S2 and S3). Contrary, the R7–R6 comparison displayed the lowest total number of redundant DEGs (353), while R4–R3 exhibited the smallest number of non-redundant DEGs (35) (Figure 1C,D). The data showed that there were a large number of genes involved in transcriptional reprogramming during salinity, most notably at 24 h–48 h of stress, confirming the previous physiological results where RUG accumulated higher amounts of the photosynthetic pigments (total chlorophyll–Chl-T and chlorophyll a–Chl-a) and the soluble sugar (e.g., total soluble sugar (TSS), glucose (Glu), fructose (Fru), and sucrose (Suc)), but ROS-related compounds at 48 h only (mainly proline and SOD) [21].

### 2.2. Cluster-Based Analysis Showed Dynamic Transcript Abundance during Salinity

**Figure 2:**
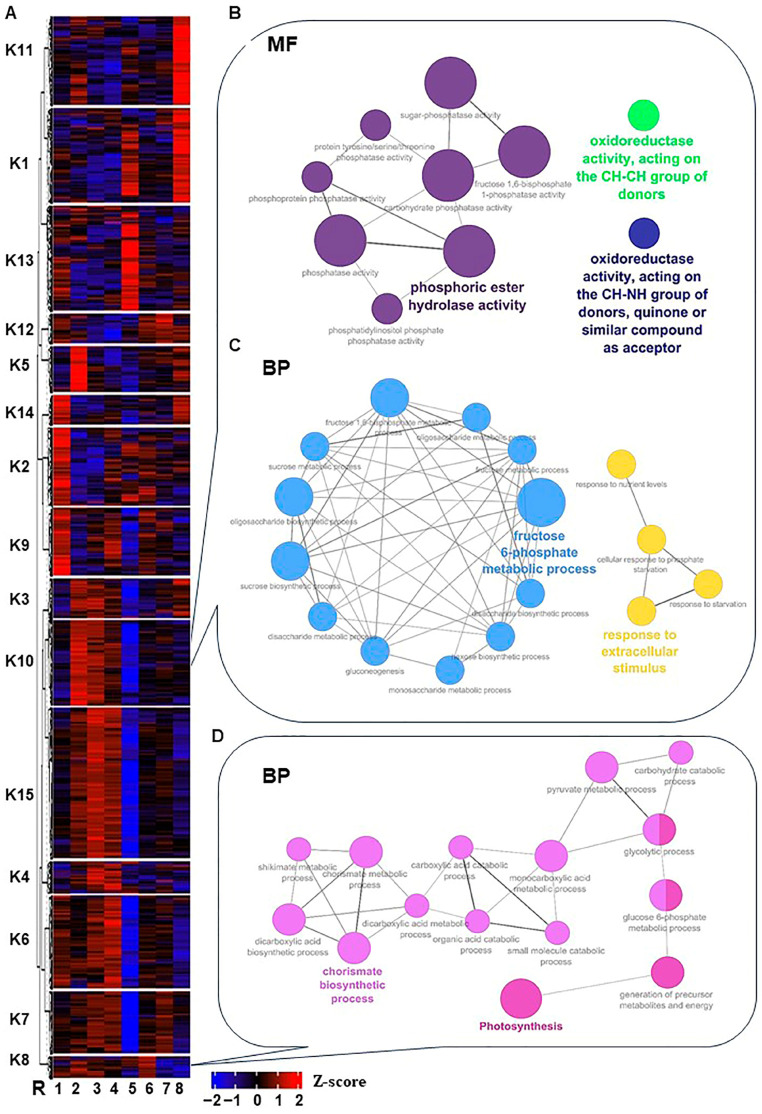
Heatmap of the K-means clusters of the 12,760 non-redundant significantly expressed genes throughout salinity stress in RUG. (**A**) By using transcript per million (TPM) of the 12,760 non-redundant significantly expressed genes during salinity stress, the K-means algorithm generated 15 clusters (K1 to K15) that were hierarchically clustered utilizing Pearson pairwise correlation. The sampling order R1–R8 refers to the sampling time, as described in Figure 1. (**B**–**D**) Network views for specific predefined Biological processes GO terms pathways for significantly expressed genes within K8 and K10, and the Molecular Function (MF) in K10 only. All MF and BP GO terms for each cluster (*p*-adjusted < 0.05) were extracted by the g:Profiler website with the Benjamini-Hochberg FDR multiple testing correction method. The default ClueGO settings were applied, and the terms are functionally grouped based on shared genes (kappa score), shown with different colors. The size of the nodes indicates the number of mapped genes ranged from 0–5, 5–10, 10–20, 20–30, and ≥30 genes. The most significant term defines the name of the group.

To investigate the temporal pattern of significantly expressed genes throughout stress and recovery, the unsupervised clustering approach “K-means clustering” was used to partition transcripts into specific accumulation profiles. The analysis classified the 12,760 non-redundant expressed genes into 15 clusters, which could be manually fitted into six groups (Figure 2A and Figure S4 and Table S3). In all cases; however, the kinetic patterns of the transcript abundance within each group were not completely identical. The first group (I) includes clusters K5 and K10, in which transcript abundance peaked at time-0 but then declined sharply (K5) or gradually (K10). The second group (II) consists of three clusters, where transcripts continued to increase within 0.5 h–2 h of salt stress, then decreased along with stress progression. These transcripts were increased during the recovery procedure (K3) or increased 4 days post-recovery and declined after that (K7 and K15). The third group (III), composed of clusters K2, K6, and K9, exhibited an initial drop at time-0 compared to control, followed by an increase in the transcript abundance, then dropped again at 24 h (K2), or dropped earlier (K6 and K9). By contrast, clusters that showed induction between 2 h–48 h of salt stress with inconsistent patterns were placed in the fourth grope (IV), including K4, K8, and K12. The fifth group showed a sharp induction at a particular time point (24 h) as in K1 and K13; however, K1 exhibited a second increase at 12 days of recovery. Finally, clusters K11 and K14 with inconsistent patterns were placed in the sixth group (IV).

To provide a broad overview of the types of transcripts in each group, the Gene Ontology (GO; i.e., Molecular Function MF, Cellular Component CC, and Biological Process BP) terms and Kyoto Encyclopedia of Genes and Genomes (KEGG) enrichment strategies were used to analyze each cluster [27]. Several stress- and salinity adaptation-related GO-term categories displayed high enrichment with a wide range of cluster differences. For instance, cluster K10 was enriched in ROS homeostasis MF subcategories (“oxidoreductase activity, acting on the CH-CH group of donors” and “oxidoreductase activity, acting on the CH-NH group of donors and quinone or similar compound as acceptor”) (Figure 2B and Figure S5 and Table S4). Moreover, K10 with K8 clusters showed considerable enrichment of BP GO terms for signaling, hormones, photosynthesis, carbohydrates, and other stress-related terms (e.g., cell communication, intracellular signal transduction, signal transduction, abscisic acid-activated signaling pathway, polyamine biosynthetic process, fructose 6-phosphate metabolic process, sucrose biosynthetic process, oligosaccharide biosynthetic process, fructose 1,6-bisphosphate metabolic process, fructose metabolic process, carbohydrate derivative metabolic process, disaccharide biosynthetic process, carbohydrate metabolic process, oligosaccharide metabolic process, monosaccharide metabolic process, disaccharide metabolic process, protein dephosphorylation, sucrose metabolic process, gluconeogenesis, oxidative photosynthetic carbon pathway, and photosynthesis) (Figure 2C,D and Figures S6 and S7 and Table S4).

The previous data analysis showed the global significant changes in transcriptome profile and, subsequently, the GO term enrichments that eventually differentiated in response to salt stress. Most notably, the photosynthesis, carbohydrates, and ROS homeostasis-related GO terms were overrepresented with the progression of salinity.

### 2.3. Analysis of Expression Patterns and Identification of WGCNA Modules Associated with Salinity Adaptation

In our previous study, we examined the physiological responses of RUG rootstock to salinity, including photosynthetic pigments (chlorophyll “Chl-T, Chl-a, Chl-b”, and carotenoids “Caro”) and sugar contents (total soluble sugar “TSS”, sucrose “Suc”, glucose “Glu”, and fructose “Fru”) as a manifestation of the efficacy of photosynthesis and sugar/energy metabolism. In addition, the enzymatic antioxidant activities of superoxide dismutase “SOD”, catalase “CAT”, and glutathione peroxidase “GPX” along with the determination of proline “Pro” content as an indication of redox homeostasis and two oxidative stress markers (malondialdehyde “MDA”, hydrogen peroxide “H_2_O_2_”) were assessed in the same samples used in this study [21]. The physiological results and the transcriptomic data suggested that RUG maintains efficient photosynthesis and antioxidative machinery under salinity by accumulating high amounts of sugars and suppressing the induction of ROS, respectively. The WGCNA system biology approach was exploited to investigate the gene modules and physiological data to get a better understanding of the molecular events under salt stress. However, as the recovery treatment alleviates the impact of salinity, its data was omitted from this analysis.

**Figure 3:**
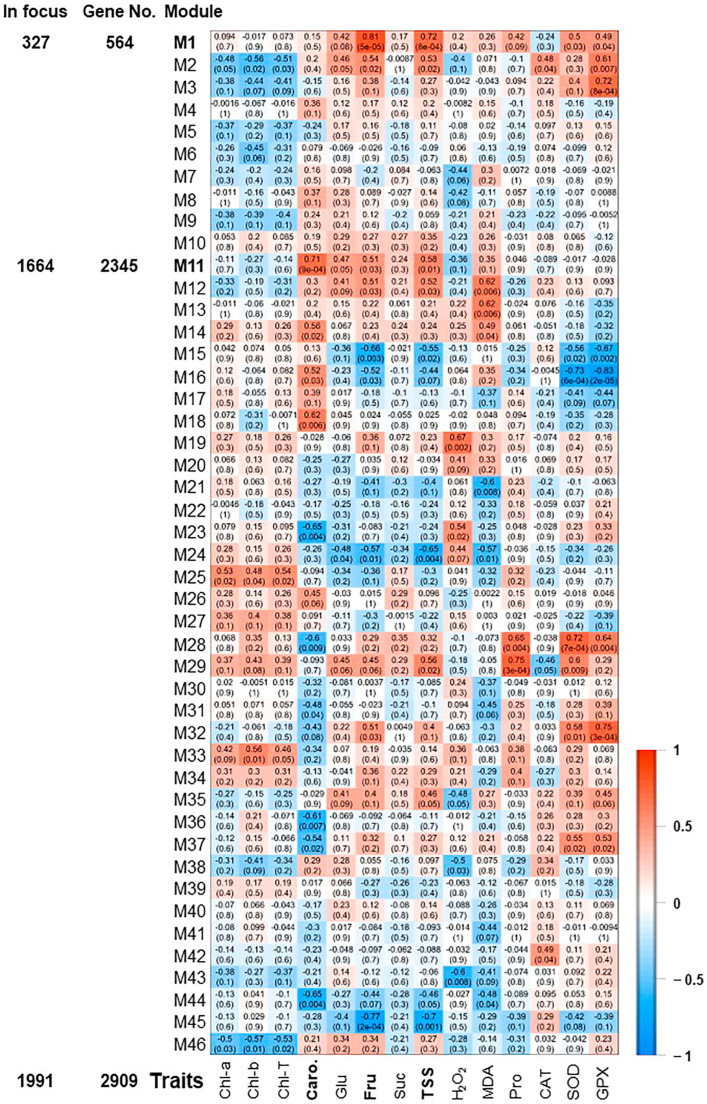
Expression heatmap of co-expression modules. Module-trait associations between RNA-seq data and evaluated physiological traits of photosynthetic pigments (chlorophyll “Chl-T, Chl-a, Chl-b”, and carotenoids “Caro”), sugar contents (total soluble sugar “TSS”, sucrose “Suc”, glucose “Glu”, and fructose “Fru”), antioxidant enzymatic activities of superoxide dismutase “SOD”, catalase “CAT”, and glutathione peroxidase “GPX”, and antioxidant non-enzymatic activity represented by proline “Pro” content [21]. The color of the cell indicates the correlation coefficient between a given module and the applicable trait at the row-column intersection. Each row corresponds to a module (M1–M46). Modules of interest (M1 and M11) were shown in bold-line and were selected for further analysis. The left panel displayed the assigned number of genes to two selected modules, whether the number of total input genes or significantly expressed genes from seven pairwise transcriptome comparisons between each two consecutive time points during salinity (R2–R1, R3–R2, R4–R3, R5–R4, R6–R5, R7–R6 or R8–R7).

The pairwise correlations among the 18 samples (control, 0 h, 0.5 h, 2 h, 24 h, and 48 h) identified 46 modules, which were labeled in distinct colors shown in a hierarchical clustering dendrogram and network heatmap (Figures S8 and S9). The analysis of module-trait correlations among the identified modules and the physiological data enabled us to identify 6 and 2 modules with positive and negative correlations, respectively (Figure 3). Among the positively correlated modules, the M1 module was highly correlated with Fru and TSS modules (r^2^ ≥ 0.81 and 0.72, respectively), holding 327 significant genes. The M3 and M32 (10 and 18 significant genes, respectively) displayed a substantial positive correlation with GPX (r^2^ ≥ 0.72). Finally, the M11, M28, and M29 modules involving 1664, 17, and 29 significant genes were positively correlated with Caro, SOD, and Pro (r^2^ ≥ 0.71), respectively. On the other hand, M16 module showed a negative correlation with SOD and GPX (r^2^ ≥ −0.73), while M45 was negatively associated with Fru (r^2^ ≥ −0.77).

**Figure 4:**
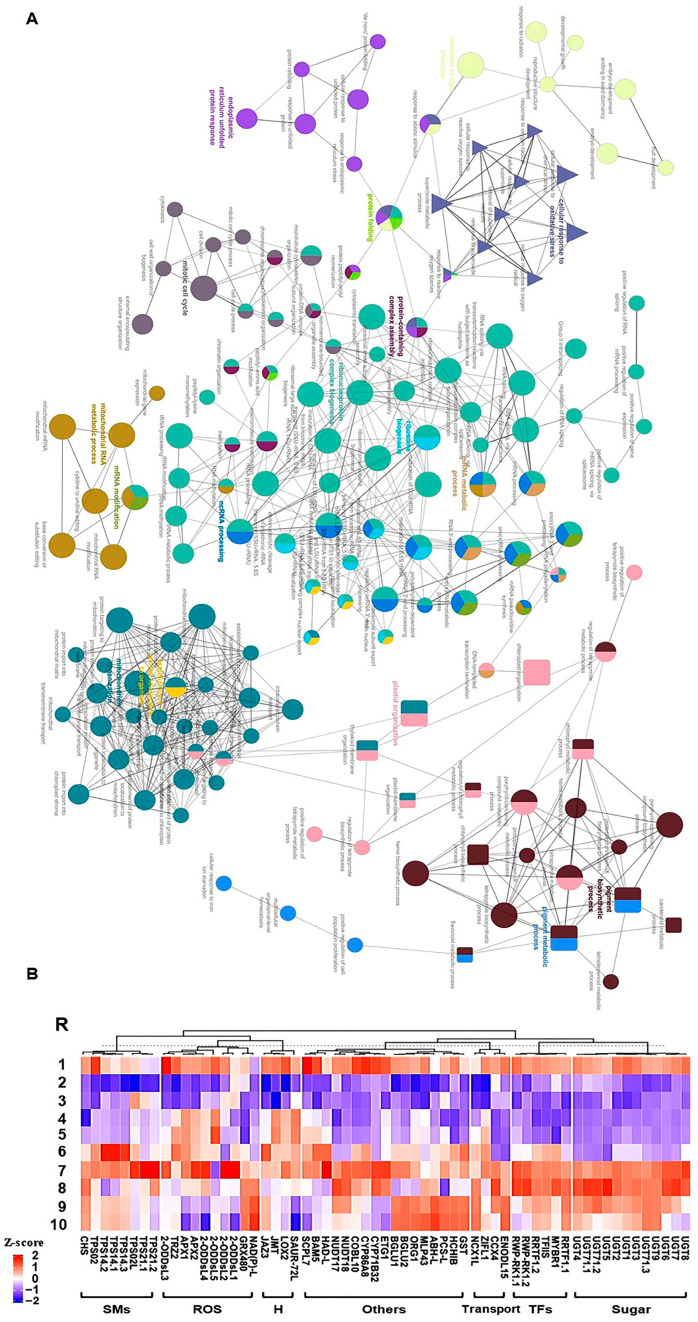
(**A**) Network views for specific predefined Biological processes GO terms pathways for significantly expressed genes within WGCNA module M11 with *p*-adjusted < 0.05. The GO terms were extracted by g:Profiler website with the Benjamini-Hochberg FDR multiple testing correction method. The default ClueGO settings were applied, and the terms are functionally grouped based on shared genes (kappa score), shown with different colors. The size of the nodes indicates the number of mapped genes ranged from 0–5, 5–10, 10–20, 20–30, and ≥30 genes. The most significant term defines the name of the group. (**B**) Heatmap of the 61 hub genes significantly expressed throughout salinity and recovery in RUG using qPCR gene expression data. Genes were hierarchically clustered using Pearson pairwise correlation. The sampling order R1-R10 refers to the sampling time as follows: control, 0 h, 0.5 h, 2 h, 8 h, 24 h, 48 h, 4 d, 8 d, and 12 d, respectively.

Consequently, we focused on the most two prominent modules (M1 and M11), exhibiting positive correlation and holding 2065 significant genes (Figure 3). The GO terms and KEGG enrichment analysis of M1 and M11 modules exposed high enrichment of GO terms associated with stress adaptation (Table S5). For instance, M1 displayed considerable enrichment of BP GO terms for “cellular response to hormone stimulus”, “hormone-mediated signaling pathway”, “ethylene-activated signaling pathway”, “regulation of biosynthetic process”, “regulation of cellular metabolic process”, “regulation of primary metabolic process”, glycosyltransferase activity, and “cell wall polysaccharide metabolic process” (Figure S10 and Table S5). However, M11 was highly enriched for BP GO terms for “plastid organization”, “chloroplast organization”, “pigment metabolic process”, “pigment biosynthetic process”, “cellular response to oxidative stress”, “chlorophyll metabolic process”, “superoxide metabolic process”, “chlorophyll biosynthetic process”, “protein import into chloroplast stroma”, “thylakoid membrane organization”, “plastid membrane organization”, “response to superoxide”, “removal of superoxide radicals”, “cellular response to superoxide”, “cellular response to oxygen radical”, “response to oxygen radical”, “regulation of chlorophyll metabolic process”, “flavonoid metabolic process”, “regulation of cellular biosynthetic process”, “carbohydrate metabolic process”, “response to reactive oxygen species”, “regulation of biosynthetic process”, and “carotenoid metabolic process” (Figure 4A and Figure S11 and Table S5). Altogether, the WGCNA approach and the GO term enrichments analysis of positively correlated modules (M1 and M11) confirmed the high correlation of photosynthesis, carbohydrates, and ROS homeostasis with salinity adaptation of RUG rootstock.

### 2.4. Identification of Hub Genes Regulating Salinity Adaptation

To identify essential genes involved in salinity adaptation, annotation information of significantly expressed genes within M1 and M11 modules was extracted from the *V. vinifera* PN40024 grapevine reference genome [28,29]. A total of 61 genes were identified and classified into 7 groups based on their predicted functions as follows: ROS detoxification, sugars metabolism, hormones, transcription factors (TFs), secondary metabolites, transport, and other functions (Figure 4B). Despite that they were exclusively located in 2 WGCNA modules, they exhibited diverse expression profiles (K1, K2, K8, K9, K11, K12, 13, K14, or no cluster) that was validated by quantitative real-time PCR (qPCR) assay. In addition to the biological samples used for transcriptome analysis, two extra points were included in the qPCR assay at 8 h of salinity and 8 days of recovery. The qPCR results of hub genes exhibited a significant correlation with mRNA-seq data (r^2^ ≥ 0.90), except for one gene that showed r^2^ ≥ 0.73, validating the transcriptome profiles (Figure 4B and Table S6).

For ROS detoxification-related genes, 9 out of 10 transcripts started to accumulate early after 0.5 h of salinity, including genes encoding ascorbate peroxidase (*APX1* and *2*) and seven oxidoreductase superfamily protein (*2-ODDsL* (5 isoforms), *NAD(P)-L*, *TRZ*). Only *GRX480*, which encodes thioredoxin superfamily protein, was considerably induced after 24 h of stress. With few exceptions, the expression levels of these ROS detoxification-related genes reached their maximal levels after 48 h of salinity that exceeds their basal levels during typical conditions; however, many of them tended to decline during the recovery procedure. For genes related to sugar metabolism, 12 transcripts encoding UDP-glycosyltransferases (*UGTs*) superfamily proteins were identified. Generally, their strong induction took place after 48 h of salt stress with a tendency to accumulate during the recovery process, establishing a peak at 4 days. Regarding the hormone-related genes, 3 out of 4 genes were jasmonic acid (JA)-related genes, while the fourth gene belongs to auxin. Except for the JA carboxyl methyltransferase (*JMT*), they showed fast accumulation during early salt stress (0.5 h). Of particular interest is the jasmonate-zim-domain protein 3 (*JAZ3*), which negatively affects the JA signal, playing a crucial role in fine-tuning the JA pathway [30,31]. Similarly, the gene encoding the SAUR-like auxin-responsive protein family (*SAUR72*) was strongly expressed between 2–24 h of stress but reached around null expression at 8–12 days of recovery.

For TF-related genes, the 6 hub genes located in module M1 showed their strong expression within 48 h of stress and 4 days of recovery compared to control, including the redox responsive transcription factor 1 (*RRTF1*, two isoforms), myb domain protein r1 (*MYBR1*), plant regulator RWP-RK family protein (*RWP-RK1*, two isoforms), E2F target gene 1 (*ETG1*), and transcription elongation factor (*TFIIS*) family protein. Genes related to secondary metabolites accumulated mainly during late stress (24–48 h). In addition to one gene for chalcone and stilbene synthase family protein (*CHS*), this set of genes mainly includes terpene synthases (*TPSs*, 7 isoforms), which are liable for the structural diversity of the superfamily of terpenoid products (also referred to as terpenoids or isoprenoids) with diverse expression profiles that decline during the recovery. For transport-related genes, four hub genes displayed distinct accumulation patterns, two of which are sodium (cation)/calcium exchanger (*NCX1L* and *CCX4*), one early nodulin-like protein 15 (*ENODL15*), and one for the zinc-induced facilitator-like 1 (*ZIFL1*). Finally, for other genes involved in the salinity tolerance, 17 genes were assessed located mainly in M11 with different expression profiles, including cytochromes P450 (*CYP71B32* and *CYP86A8*), nudix hydrolases (*NUDT17* and *18*), major latex protein-like protein 43 (*MLP43*), basic chitinase (*HCHIB*), alpha/beta hydrolase (ABH) superfamily (*ABH-L*), serine carboxypeptidase-like 7 (*SCPL7*), beta-amylase (*BGLU1*, *BGLU2*, and *BAM5*), halo acid dehalogenase (HAD) superfamily (*HAD-L*), glutathione S-transferase (*GST*), COBRA-like protein 10 precursor (*COBL10*), Eukaryotic aspartyl protease family protein (*PCS-L*), and the OBP3-responsive gene 1 (*ORG1*).

## 3. Discussion

In this study, leaves of RUG grapevine rootstock showed transcriptome reprogramming profiles over salinity and recovery conditions. Additional levels of complication arose from the experimental procedure with different time points of both salinity and recovery. Moreover, the re-watering application counteracted salinity, alleviating its adverse effects on transcriptome, as shown by PCA results. Monitoring the growth status of grapevine plants indicated that this influence is stress-dependent, and significantly impacted by its gravity and duration [32]. In addition, the genetic background of plants determines the effect of salinity and, accordingly, the transcriptome remodeling as shown by the coastal salt-tolerant wild grapevine AS1B compared to salt-sensitive rootstock Richter 110 [33]. The pairwise comparisons between transcriptome profiles of consecutive time points of RUG during salinity and recovery showed substantial changes in its transcriptomic profiles. Notably, when the 24 h of stress was compared against the 2 h of stress (R5–R4), it exhibited the highest number of up- and down-regulated transcripts. Likewise, transcriptomic analysis of salt-tolerant wild grapevine Tebaba roots throughout salinity depicted similar results commensurate with the accumulation of polyphenol content and enhancement of antioxidant enzymatic activities [34]. The longer the time under salinity (in the range of 24–48 h), the higher the changes at the molecular, metabolic, and physiological levels.

It is tempted to indicate that RUG is a salt excluder rootstock with salt tolerance capacity accumulating higher levels of photosynthetic pigments and sugars along with enhanced ROS detoxification activity under salinity compared to the less tolerant Millardet et de Grasset 420A [21,23]. Therefore, we were guided by our previous results, where physiological parameters, including photosynthetic pigments, and sugar contents, along with enzymatic and non-enzymatic antioxidant activities were estimated in the same RUG samples used for the transcriptome analysis [21]. From a physiological perspective, salt-stressed plants exhibited a considerable reduction in vital biological processes such as photosynthesis, energy, and water/nutrient acquisition, but induction of ROS might lead to cell death based on stress severity and plant tolerance capacity [8,9]. The WGCNA strategy was able to identify 8 modules, exhibiting significant correlation with the recently published physiological data [21] in which 6 modules were positively correlated with carotenoids, Fru, TSS, GPX, SOD, and Pro. The most prominent two modules of M1 and M11 encompass 327 and 1664 significant genes that were highly enriched of BP GO terms related to salinity adaptation. While M1 was enriched with GO terms linked to hormones and polysaccharides, M11 was highly enriched with GO terms for photosynthesis, ROS detoxification, and carbohydrate metabolic processes. However, the K-means clustering showed that each module could include different clusters with distinct kinetic behaviors and, thus, a wide range of GO terms and KEGG enrichment analysis for each cluster.

The considerable enrichment of BP GO terms for signaling, hormones, photosynthesis, carbohydrates, ROS homeostasis, and other stress-related terms in M1 and M11 modules suggested a battery of molecular/cellular responses established during salinity. Certainly, salt-challenged plants accumulate high inorganic ions, especially Na^+^ and Cl^−^, resulting in cellular toxicity, nutritional and energetic imbalances, and lipid peroxidation. The stress ultimately leads to the production of ROS and metabolic dysfunction, which impair photosynthesis and nutrient acquisition, causing cell death based on stress severity [2,3,4]. Therefore, the identified 61 hub genes were selected to cover a wide range of salt-adaptation processes, including ROS detoxification, sugar metabolism, hormones, transcription factors (TFs), secondary metabolites, transport, and other functions. Salinity is generally accompanied by overproduction of ROS and alterations in secondary metabolite accumulation [35]. Hence, plants have to swiftly re-establish their ROS homeostasis. Our results showed that 9 out of 10 ROS detoxification-related genes started accumulating earlier after 0.5 h of salinity, such as *APX1/2* and seven genes encoding oxidoreductase superfamily protein. The role of the thioredoxin superfamily and *APX* in redox homeostasis is not negotiable [36]. For example, APX is an antioxidant enzyme that functions in synthesizing ascorbate (AsA) and scavenging the H_2_O_2_. Ectopic expression of Celery (*Apium graveolens*) *APX1* into *Arabidopsis* resulted in the induction of AsA content and antioxidant capacity but less reduction in photosynthetic rate and chlorophyll content that culminated in drought resistance [37].

Interestingly, other gene-encoded proteins from different pathways also have ROS detoxification activity besides their primary function. For instance, the UDP-glycosyltransferases (*UGTs*) and terpene synthases (*TPSs*) have a crucial role in detoxification and homeostasis besides their prominent role of catalyzing a sugar conjugation with small lipophilic compounds and generating the structural diversity of terpenoids superfamily, respectively [38,39]. Further, terpenes and terpenoids have a wide range of biological activities, acting as anticancer, antimicrobial, anti-inflammatory, and anti-allergic [39]. Similarly, TFs such as *RRTF1* and *MYBR1* were up-regulated by stress and H_2_O_2_, resulting in less ROS accumulation due to efficient antioxidant machinery [40,41]. On the contrary, the TFs of *RWP-RK* play essential roles in nitrate starvation responses and root nodulation [42], while the activity of TFIIS is substantial for heat stress adaptation in plants, while its absence negatively affects the expression and the alternative splicing pattern of hundreds of heat-regulated transcripts [43].

ROS can highly coordinate and regulate multiple signaling pathways such as antioxidants, kinases, defense-related genes, Ca^2+^ influx, and hormones (e.g., JA, ethylene, and salicylic acid) [2,44]. Our data showed that transport-related genes encoding cation/Ca^2+^ exchanger were strongly induced during late stress and recovery. Similarly, the *ZIFL1* is critical in regulating stomatal closure during stress in *Arabidopsis* [45]. Moreover, the *ENODL* genes, such as *GhENODL6*, were shown to exhibit SA-induction under biotic stress and regulate the ROS production in cotton [46]. Furthermore, 3 out of 4 hormone-related genes are JA-related, especially the *JAZ3* that showed strong induction at 2–48 h compared to 0 h. It reported that salinity adaptation in grapevine is highly correlated with tight control of JA and its isoleucine conjugate (JA-Ile) production and, hence, fine-tuning their signals [3]. In contrast, salt sensitivity is more likely attributed to the unconstrained jasmonate (JA) signaling accompanied by ROS burst. Finally, our results revealed a list of genes with different functions that are up-regulated under salinity with involved stress tolerance and antioxidant activities during stress such as cytochromes P450, nudix hydrolases, major latex protein-like protein, serine carboxypeptidase-like, and glutathione S-transferase [47,48,49,50,51].

In conclusion, this investigation addressed the transcriptional remodeling of grapevine rootstock RUG throughout salinity and recovery, complementing our previous physiological study [21] by improving our understanding of the molecular dynamic coordinating grapevine’s salt tolerance mechanism. The data demonstrated the significant impact of establishing different sets of processes to cope with salinity stress, most notably ROS detoxification and homeostasis. Future research would allow further in-depth validation and functional characterization of pivotal stress biomarkers to select suitable traits for generating innovative salt-tolerant rootstocks.

## 4. Materials and Methods

### 4.1. Plant Materials, Salinity Application, and Sampling

Leave samples were collected from 3-year-old *Vitis* hybrid 140 Ruggeri (*V. berlandieri* × *V. rupestris*) grown under greenhouse conditions during the summer season of 2020 at the Florida A&M University (Tallahassee, FL, USA). The hybrid rootstock was selected according to its salinity adaptation [21,22]. A total of 50 grapevine rootstock plants were used for the experiment through which each plant was grown in a one-gallon pot supported with a based plate and peat moss mixed with perlite (3:1). Salt stress was applied gradually by irrigating plants with 2.5 L of 30 mM NaCl daily for 5 days to reach 150 mM NaCl as a final concentration. Once the fifth addition of NaCl was received, samples were collected from mature and healthy leaves, mainly the third and/or fourth ones. At each time point, fifteen leaves were collected and distributed into 3 biological replicates (5 leaves/replicate). For the salinity time course, leave samples were collected at time points 0, 0.5, 2, 8, 24, and 48 h. Control plants were irrigated using distilled water. Similarly, treated plants were re-watered with distilled water for the recovery experiment, and leaf samples were collected at 4, 8, and 12 days. All samples were immediately flash-frozen in liquid nitrogen and stored at −80 °C for further analysis.

### 4.2. Nucleic Acid Extraction and RNA-Seq Library Construction

Total RNA was extracted from leaf samples as described previously [52]. All RNA samples were treated with the RNase-Free DNase Set (Qiagen, Valencia, CA, USA), then cleaned up with the RNeasy Mini Kit (Qiagen). A total of 24 RNA-seq libraries (three biological replicates at 8 salt-stressed time points, R1–R8) were constructed using NEBNext Ultra II RNA Library Prep Kit for Illumina (New England Biolabs, Ipswich, MA, USA). The libraries were multiplexed equally for paired-end 150-base sequencing in two lanes of NovaSeq 6000 (Illumina, San Diego, CA, USA) at the Novogene Co., Ltd. (Sacramento, CA, USA).

### 4.3. RNA-Seq Data Preprocessing and Identification of Differentially Expressed Genes

Illumina sequencing of the multiplexed RNA-seq libraries yielded 24 FASTQ files of sequences (GenBank accession number: PRJNA1036264). As previously described [53], reads quality was examined by FastQC (https://www.bioinformatics.babraham.ac.uk/projects/fastqc/, accessed on 10 February 2023) twice before and after trimming using Trimmomatic v0.39 [54]. Trimmed reads were aligned to the *Vitis* genome (*V. vinifera*_457_Genoscope.12X) and projected to the transcriptome using STAR (Table S1) [24,25]. The later transcriptomic coordinates files (*.BAM) were then quantified by Salmon in alignment mode (Table S1) [26].

Differentially expressed genes (DEGs) during salinity and recovery times were identified between consecutive time points (R2–R1, R3–R2, R4–R3, R5–R4, R6–R5, R7–R6 or R8–R7) using DESeq2 package setting on default parameters [55]. DEGs of each comparison (DEGs, P_FDR_ < 0.05, log2fold change > ±1.5) were considered to be expressed (Table S2). The open-source R package UpSetR was used to generate a scalable matrix-based visualization for all intersections among comparisons [56]. Finally, the web-based tool Venny was used to construct the consensus result (https://bioinfogp.cnb.csic.es/tools/venny/index.html, accessed on 12 February 2023).

### 4.4. Weighted Gene Co-Expression Network Analysis

Co-expression network modules were constructed using the variance stabilizing transformation values and the R package WGCNA (v. 1.72-1) [57]. Lowly expressed genes among all samples were removed by DESeq2, and the remaining 20,717 genes were used in module construction. The co-expression modules were obtained using the default settings, except that the soft threshold power was 4, TOMType was signed, minModuleSize was 30, mergeCutHeight was 0.25, and scale-free topology fit index 0.8 (R^2^ = 0.8). A module eigengene (ME) value, which summarizes the expression profile of a given module as the first principal component, was calculated and used to evaluate the association of modules with leaf physiological parameters, including photosynthetic pigments (chlorophyll “Chl-T, Chl-a, Chl-b”, and carotenoids “Caro”), sugar contents (total soluble sugar “TSS”, sucrose “Suc”, glucose “Glu”, and fructose “Fru”), enzymatic antioxidant activities (superoxide dismutase “SOD”, catalase “CAT”, and glutathione peroxidase “GPX”), non-enzymatic antioxidant activities (proline content “Pro”), and two stress markers malondialdehyde “MDA” and hydrogen peroxide “H_2_O_2_” [21]. As a result, the final matrix for WGCNA was assigned to 46 modules (M1-M46) with 20,717 genes. The module membership (MM) and gene significance (GS) values were calculated, and the intra-modular hub genes were identified (GS > 0.2, MM > 0.8, and *p*-value < 0.05).

### 4.5. GO Enrichment and KEGG Pathway Analyses

GO and KEGG enrichment analyses were assigned using the g:Profiler website by applying the Benjamini-Hochberg multiple testing correction method with P_FDR_ < 0.05 [27]. The Cytoscape plug-in ClueGO was used to visualize the non-redundant GO terms and KEGG pathways [58].

### 4.6. Validation of DEG Subsets by qPCR

DNase-treated RNA (4 μg) was reverse transcribed in a reaction of 50 μL using the High Capacity cDNA Reverse Transcription Kit (Applied Biosystems, Foster City, CA, USA). Gene-specific primers were designed using Primer Express (v3.0, Applied Biosystems) (Table S6). The qPCR assays were performed using 20 ng of cDNA and 300 nM of each primer in a 10 μL reaction volume with SsoAdvanced Universal SYBR Green Supermix (Bio-Rad Laboratories, Hercules, CA, USA). Three biological and three technical replicates for each reaction were analyzed on a CFX384 Touch Real-Time PCR Detection System instrument (Bio-Rad Laboratories) with the first step of 95 °C for 5 min followed by 40 cycles of 95 °C for 10 s, 60 °C for 10 s, and 72 °C for 20 s. Melting curves were generated using the program: 95 °C for 15 s, 60 °C for 15 s, and 95 °C for 15 s. Transcript abundance was quantified using standard curves for the target and reference genes generated from serial dilutions of PCR products from corresponding cDNAs. Transcript abundance was normalized to the reference genes *MrActin* and *MrEF1*, which showed high stability across the different muscadine genotypes and tissues. The geometric mean of the selected housekeeping genes was validated as an accurate normalization factor.

## Data Availability

The data presented in this study are available upon request.

## Supplementary Materials

The following supporting information can be downloaded at: https://www.mdpi.com/article/10.3390/plants13060837/s1, Figure S1: Heatmap of sample-to-sample distances of RNA-seq data from Ruggeri leaves during the salt stress and recovery procedure, Figure S2: The Upset plot of the total number of the upregulated differentially expressed genes (DEGs), Figure S3: The Upset plot of the total number of the down-regulated differentially expressed genes (DEGs), Figure S4: K-means clustering of non-redundant significantly expressed genes during salt stress and recovery procedure, Figure S5: A network view for the pre-defined molecular function (MF) GO terms pathways for the K10 cluster in-group (I), Figure S6: A network view for the pre-defined Biological process (BP) GO terms and KEEG pathways for the K10 cluster in the group (I), Figure S7: A network view for the pre-defined Biological process (PB) GO terms and KEEG pathways for K8 cluster in the group (IV), Figure S8: Sample dendrogram and trait heatmap, Figure S9: The hierarchical cluster dendrogram of genes showing co-expressed modules identified by WGCNA, Figure S10: A network view for the pre-defined Biological process (BP) GO terms pathways for the WGCNA M1 module, Figure S11: A network view for the predefined Biological process (BP) GO terms pathways for the WGCNA M11 module; Table S1: Summary of read mapping rate of RNA-Seq library of Ruggeri leaves at different time points of salt stress or recovery experiment, Table S2: Significantly expressed genes over the time course of salt stress and recovery in Ruggeri rootstock, Table S3: The gene list of each K-mean cluster including their transcript per million (TPM) values and annotation information, Table S4: The GO-term information of the gene list of each K-means cluster, Table S5: The GO-term information of the gene list of each significantly correlated WGCNA module, and Table S6: The oligonucleotide primers of assessed genes by qPCR and the correlation coefficient between RNA-seq and qPCR data.
