# Supplementary material for: Transcriptome Profiling of a Salt Excluder Hybrid Grapevine Rootstock ‘Ruggeri’ throughout Salinity
Source: Plants (Basel). 2024 Mar 14;13(6):837. doi: 10.3390/plants13060837 (PMC10974295; doi:10.3390/plants13060837)
Supplement: Supplementary file 1 [file plants-13-00837-s001.zip › Supplementary Figures.pdf]

Supplementary Figures

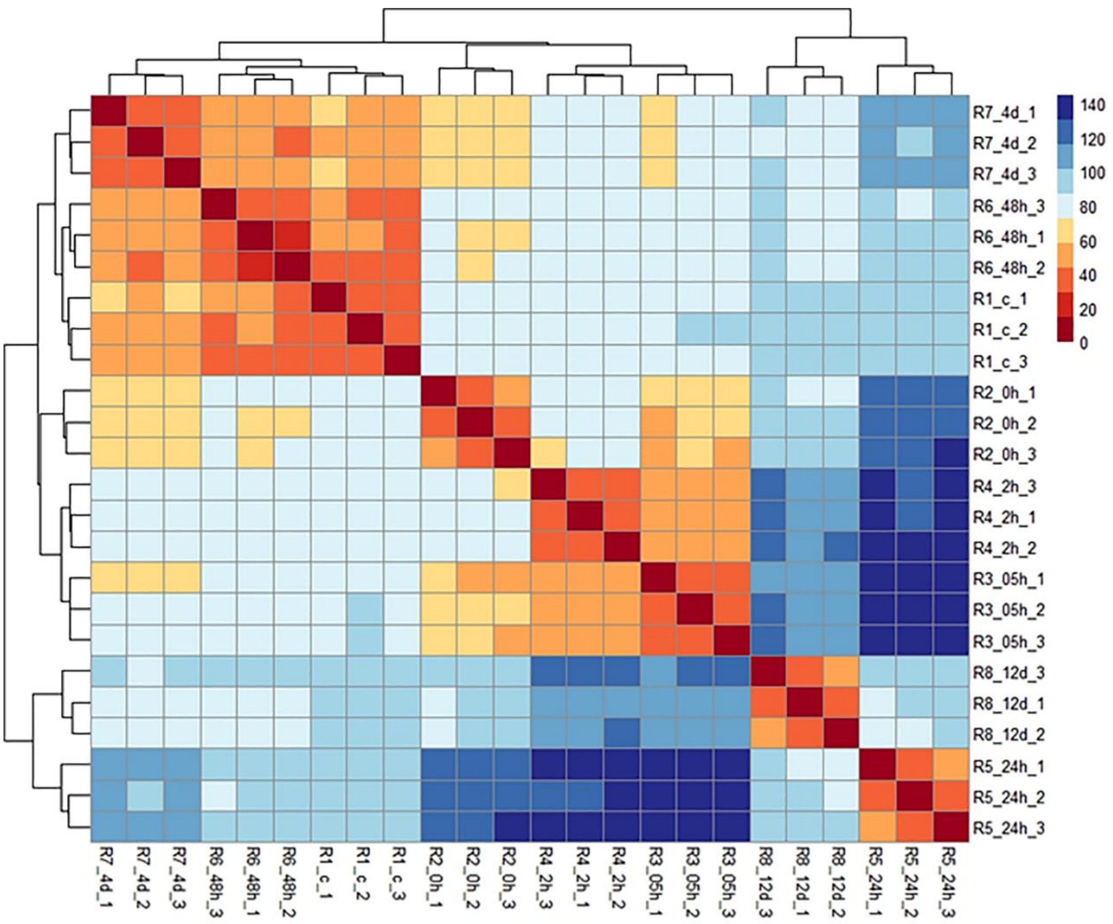

Fig. S1. Heatmap of sample-to-sample distances of RNA-seq data from Ruggeri leaves during the salt stress and recovery procedure using the variance stabilizing transformation (VST) and Euclidean distances. Sampling order R1 – R8 refers to the sampling time as follows; control, 0 h, 0.5 h, 2 h, 24 h, 48 h, 4 d, and 12 d, respectively.

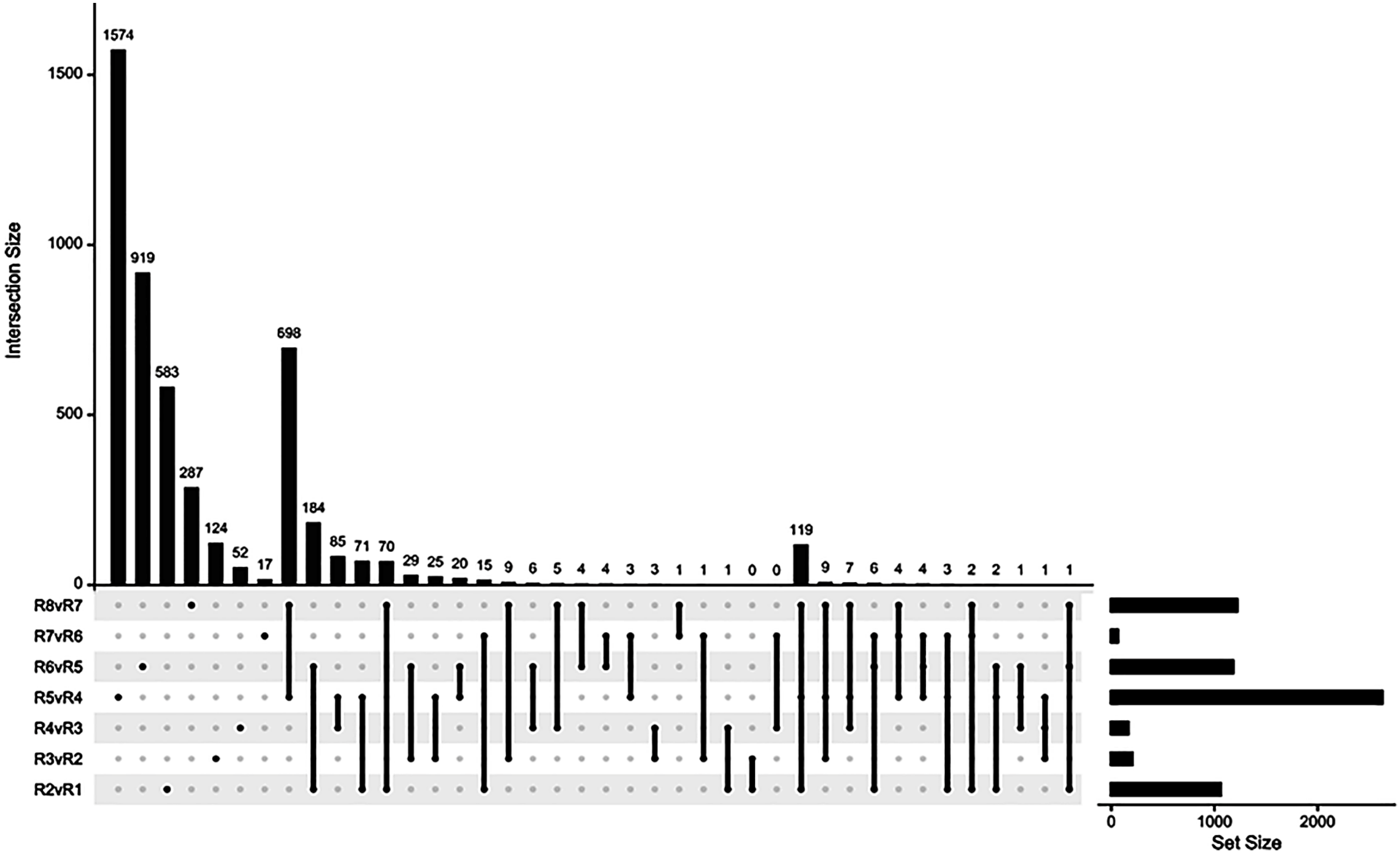

Fig. S2. The Upset plot of the total number of the upregulated differentially expressed genes (DEGs) of each comparison. By using DESeq2 pipeline, each time point during salinity and recovery procedures was compared to its former one, resulted in 6505 (4950 non-redundant), with |log2FC| ≥ ±1.5, P-adjust < 0.05. For simplicity, the sampling order 1 – 8 will be used to refer to the sampling time as follows; control, 0 h, 0.5 h, 2 h, 24 h, 48 h, 4 d, and 12 d, respectively.

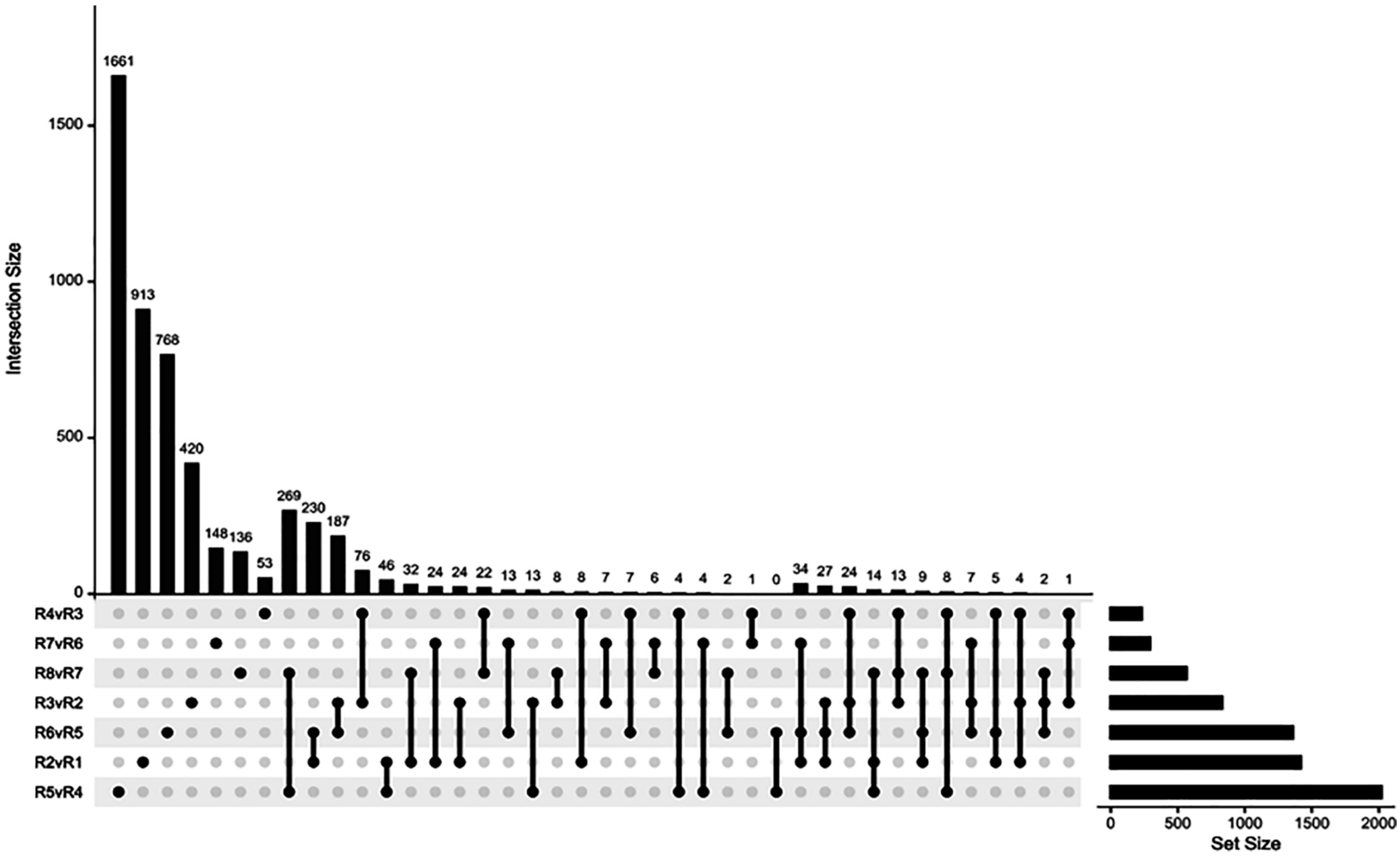

Fig. S3. The Upset plot of the total number of the downregulated DEGs of each comparison. By using DESeq2 pipeline, each time point of the course of salinity and recovery conditions was compared to its former one, resulted in 6709 (5278 non-redundant), with |log2FC| ≥ ±1.5, P-adjust < 0.05. For simplicity, the sampling order 1 – 8 will be used to refer to the sampling time as follows; control, 0 h, 0.5 h, 2 h, 24 h, 48 h, 4 d, and 12 d, respectively.

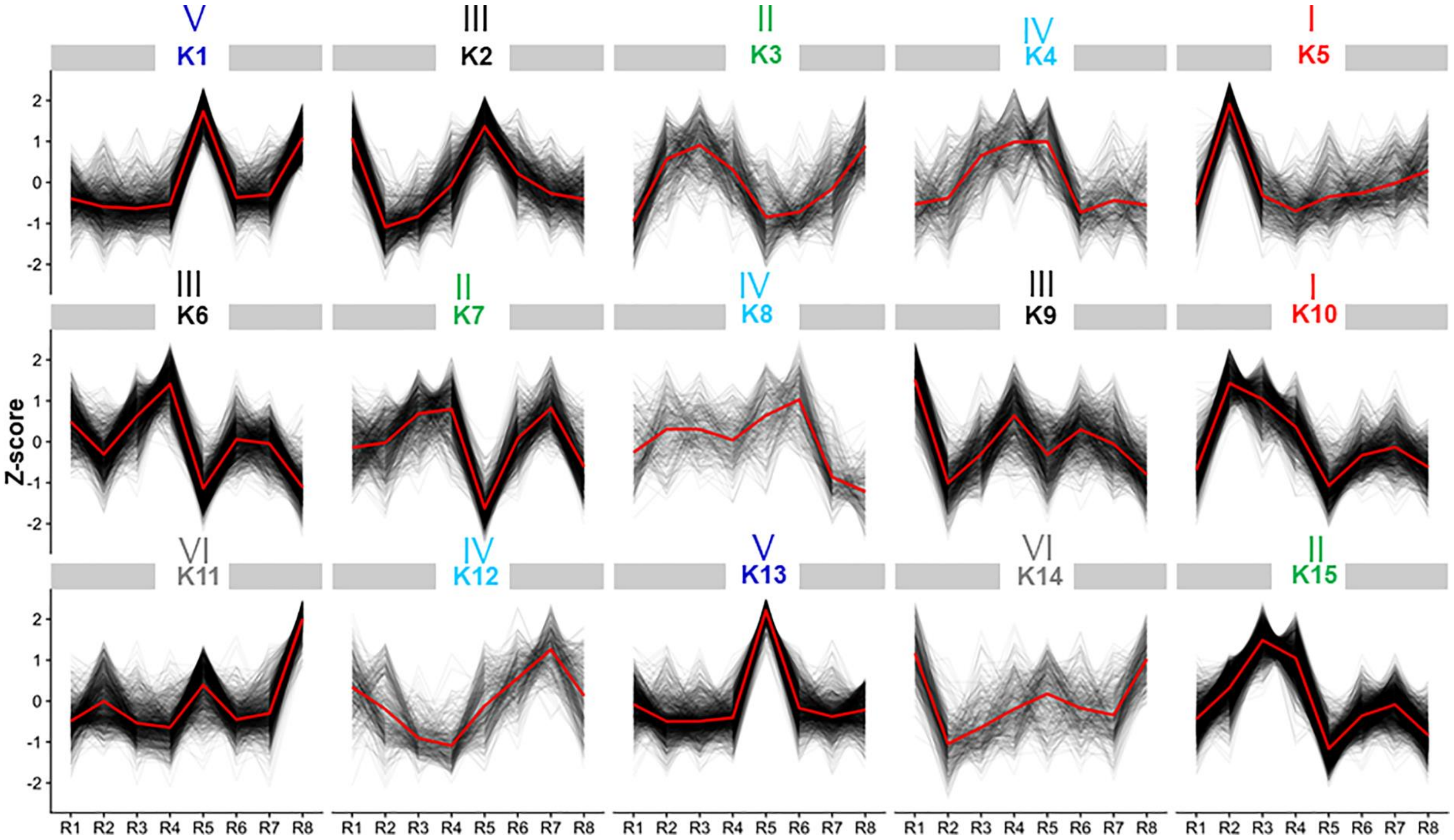

Fig. S4. K-means clustering of 12760 non-redundant significantly expressed genes during salt stress and recovery procedure. By using transcript per million (TPM), K-means algorithm generated 15 clusters (K1 to K15). The sampling order R1 – R8 refers to the sampling time as follows; control, 0 h, 0.5 h, 2 h, 24 h, 48 h, 4 d, and 12 d, respectively.

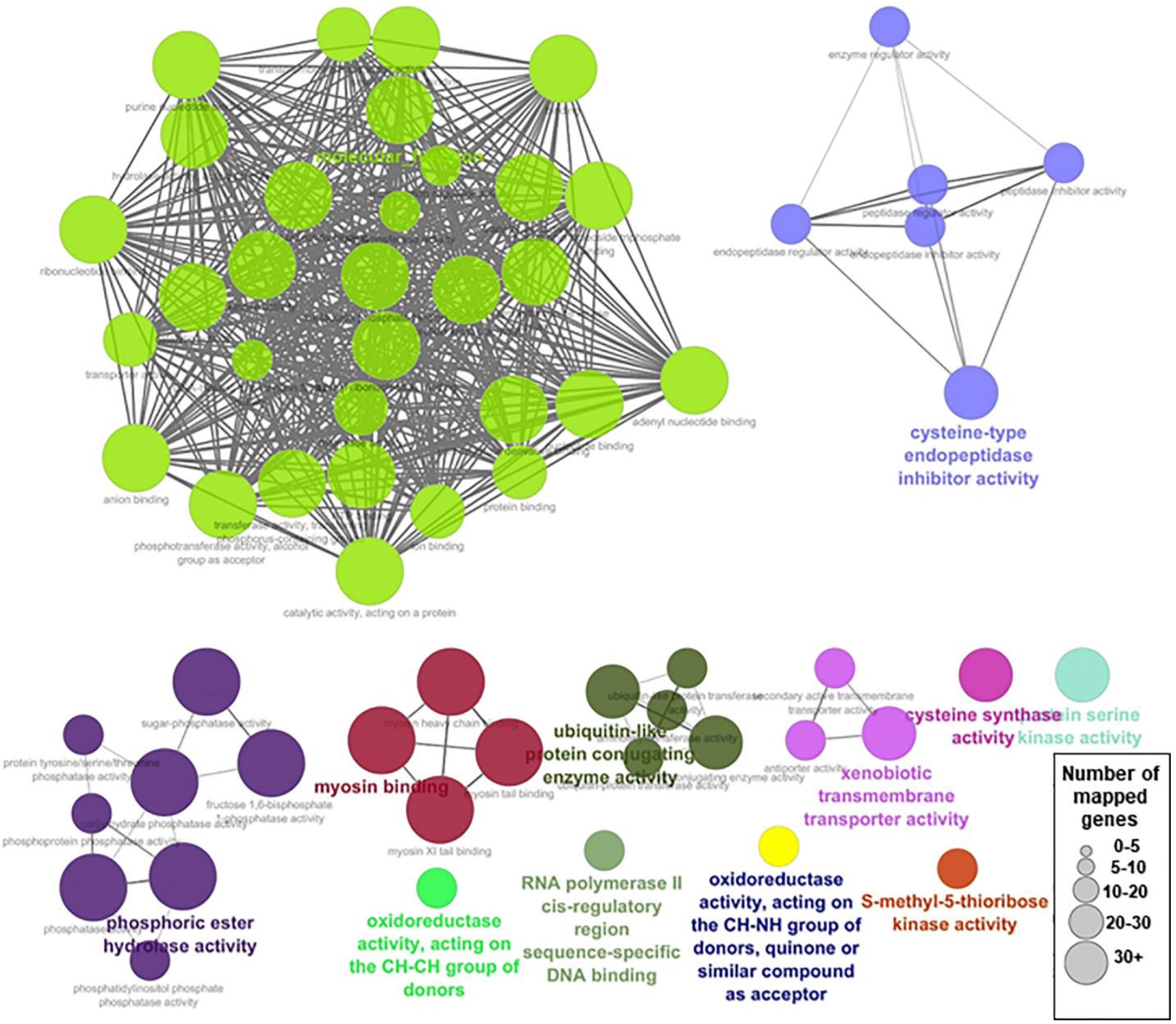

Fig. S5. A network view for the pre-defined molecular function (MF) GO terms pathways for the K10 cluster in the group (I). The MF GO terms for the K10 cluster (P-adjust <0.05) were extracted by g:Profiler website with Benjamini-Hochberg FDR multiple testing correction method. The default ClueGO settings were applied, and the terms were functionally grouped based on shared genes (kappa score). The size of the nodes indicated the number of mapped genes ranged from 0-5, 5-10, 10-20, 20-30, and ≥30 genes. The most significant term defines the name of the group.

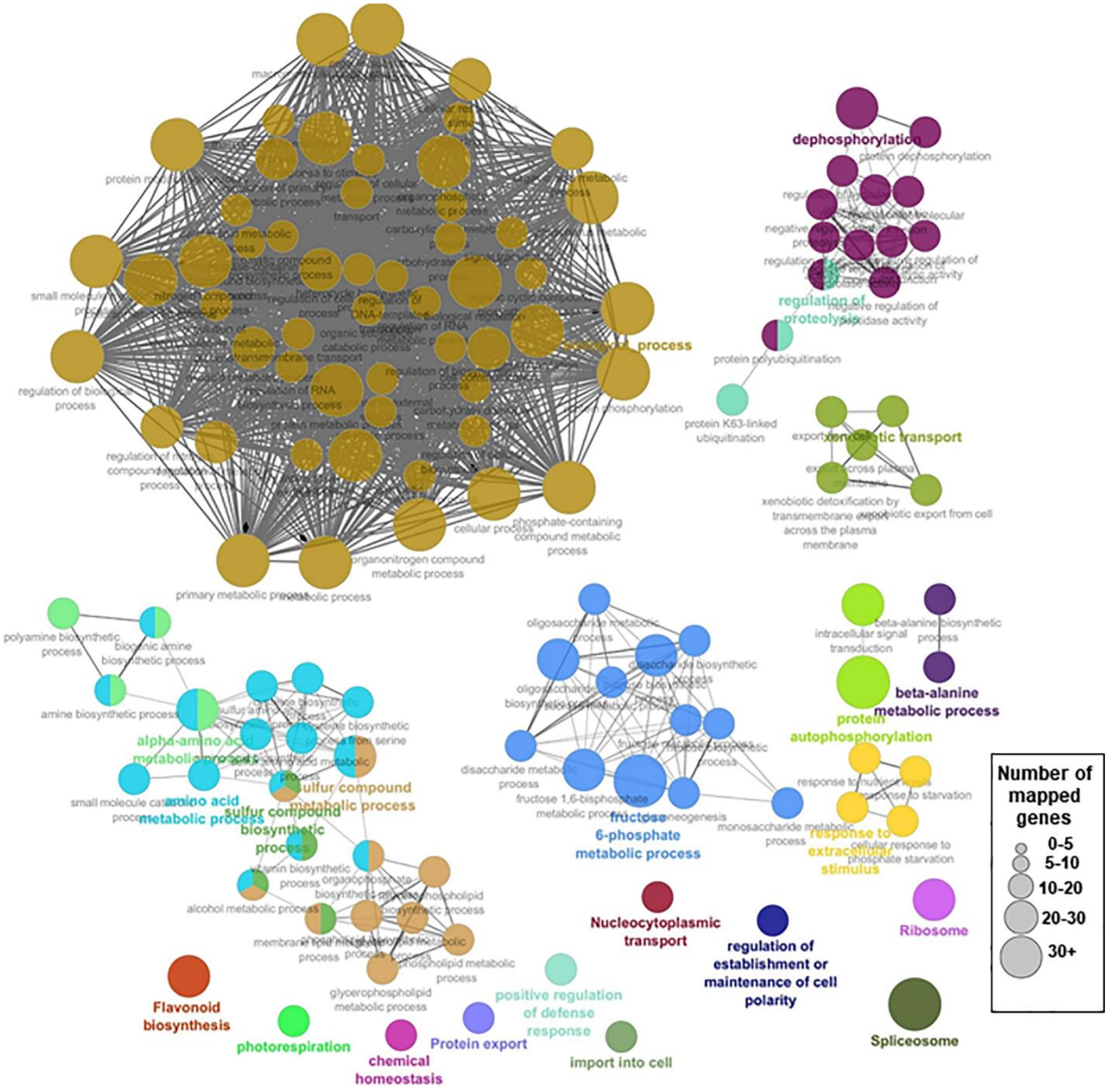

Fig. S6. A network view for the pre-defined Biological process (BP) GO terms and KEEG pathways for the K10 cluster in the group (I). BP GO terms and KEEG pathways (P-adjust <0.05) were extracted by g:Profiler website with Benjamini-Hochberg FDR multiple testing correction method. The default ClueGO settings were applied and the terms were functionally grouped based on shared genes (kappa score). The size of the nodes indicated the number of mapped genes ranged from 0-5, 5-10, 10-20, 20-30, and ≥30 genes. The most significant term defines the name of the group.

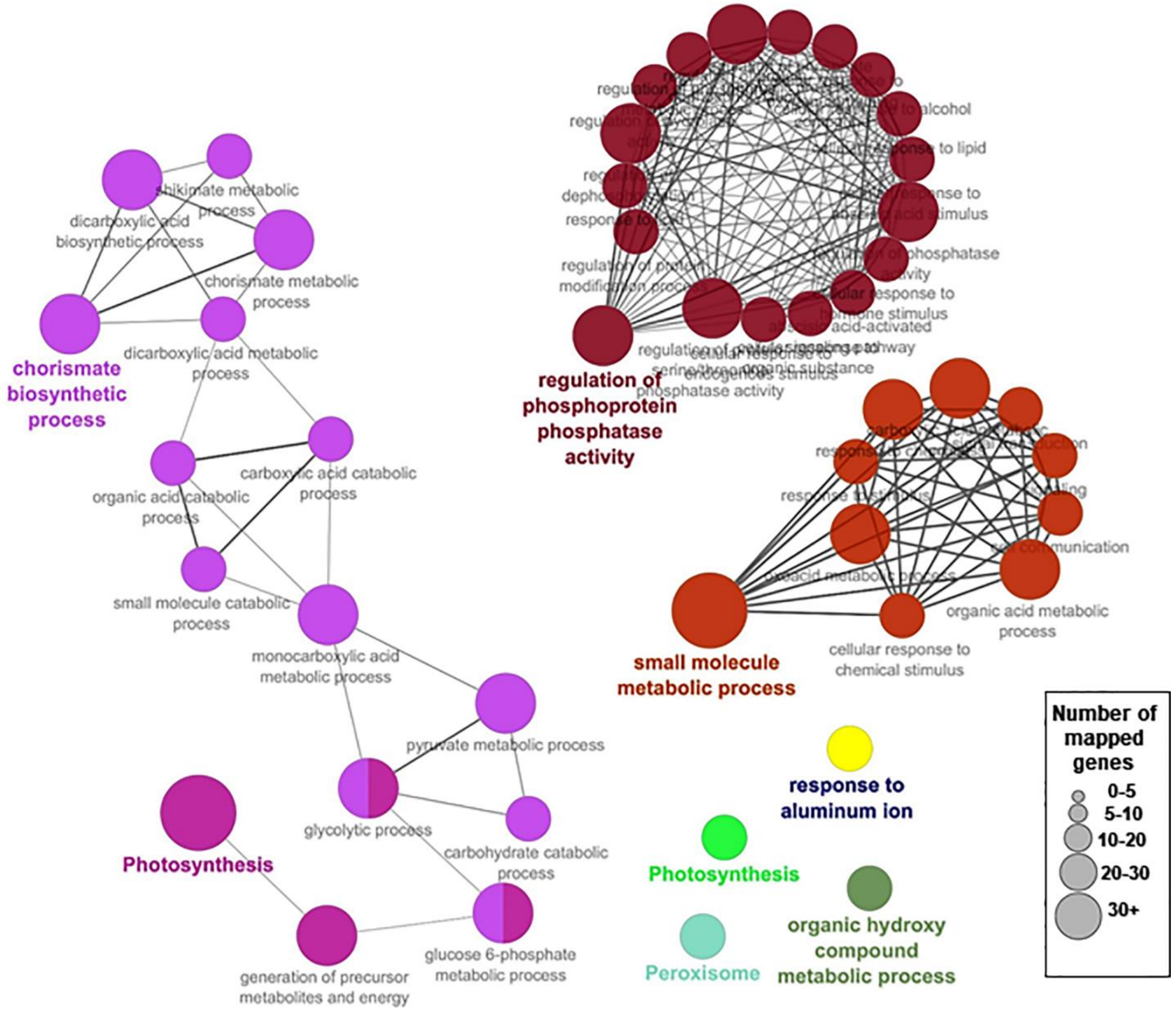

Fig. S7. A network view for the pre-defined Biological process (PB) GO terms and KEEG pathways for K8 cluster in the group (IV). The BP GO terms and KEEG pathways (P-adjust <0.05) were extracted by g:Profiler website with Benjamini-Hochberg FDR multiple testing correction method. The default ClueGO settings were applied, and the terms are functionally grouped based on shared genes (kappa score). The size of the nodes indicates the number of mapped genes ranged from 0-5, 5-10, 10-20, 20-30, and ≥30 genes. The most significant term defines the name of the group.

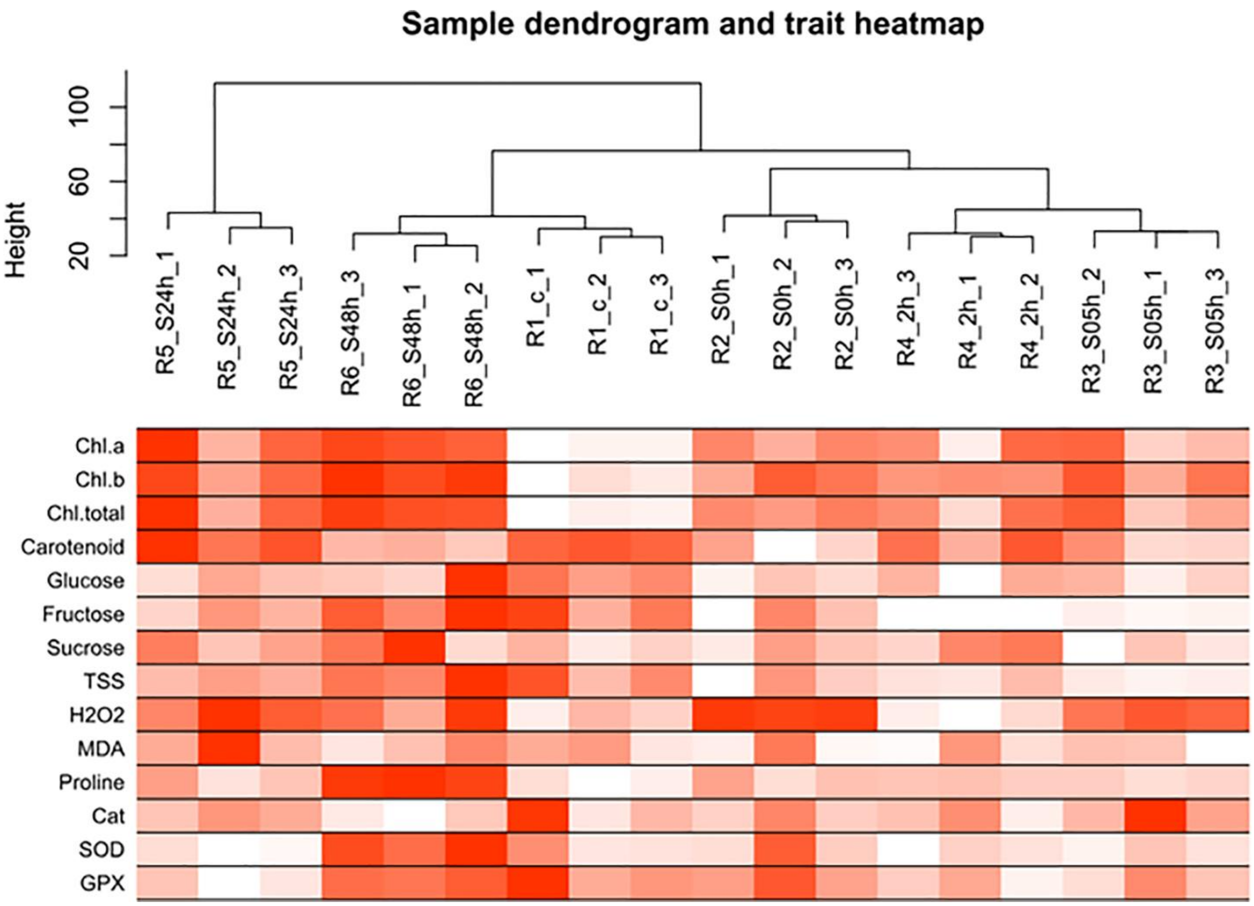

Fig. S8. Sample dendrogram and trait heatmap. Clustering dendrogram of RNA-seq samples (20717 genes) from Ruggeri leaves during the salinity procedure based on their Euclidean distance. The sampling order R1 – R6 refers to the sampling time as follows; control, 0 h, 0.5 h, 2 h, 24 h, and 48 h, respectively. White and red color represents low and high values, respectively.

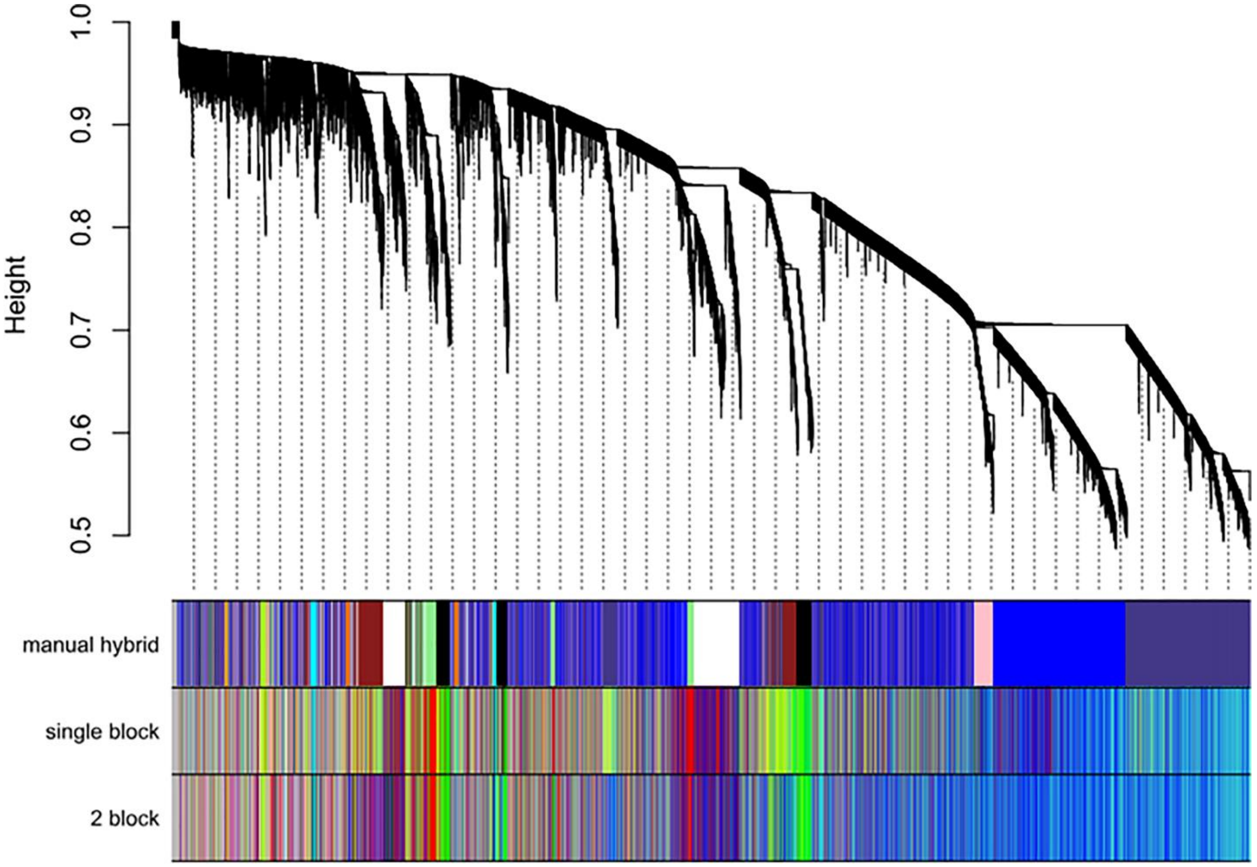

Fig. S9. The hierarchical cluster dendrogram of 20717 genes showing co-expressed modules identified by weighted gene co-expression network analysis (WGCNA) for the RNA-seq data. Each leaf on the tree is counted for one gene. The color row underneath the dendrogram shows the 46 merged module colors (based on a threshold of 0.25), as well as the original module colors, labeled with different colors.

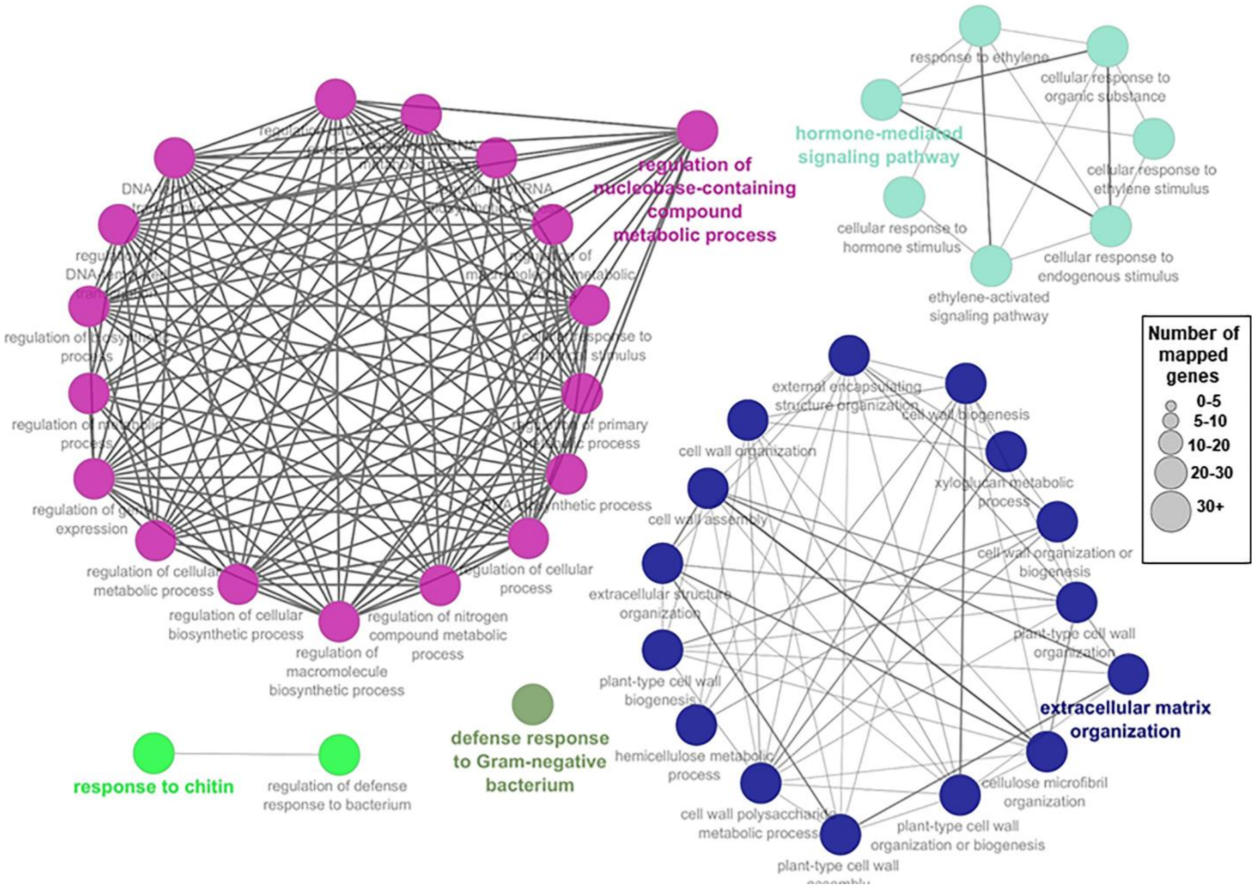

Fig. S10. A network view for the pre-defined Biological process (BP) GO terms pathways for the WGCNA M1 module. The BP GO terms pathways (P-adjust <0.05) were extracted by g:Profiler website with Benjamini-Hochberg FDR multiple testing correction method. The default ClueGO settings were applied, and the terms are functionally grouped based on shared genes (kappa score). The size of the nodes indicates the number of mapped genes ranged from 0-5, 5-10, 10-20, 20-30, and ≥30 genes. The most significant term defines the name of the group.

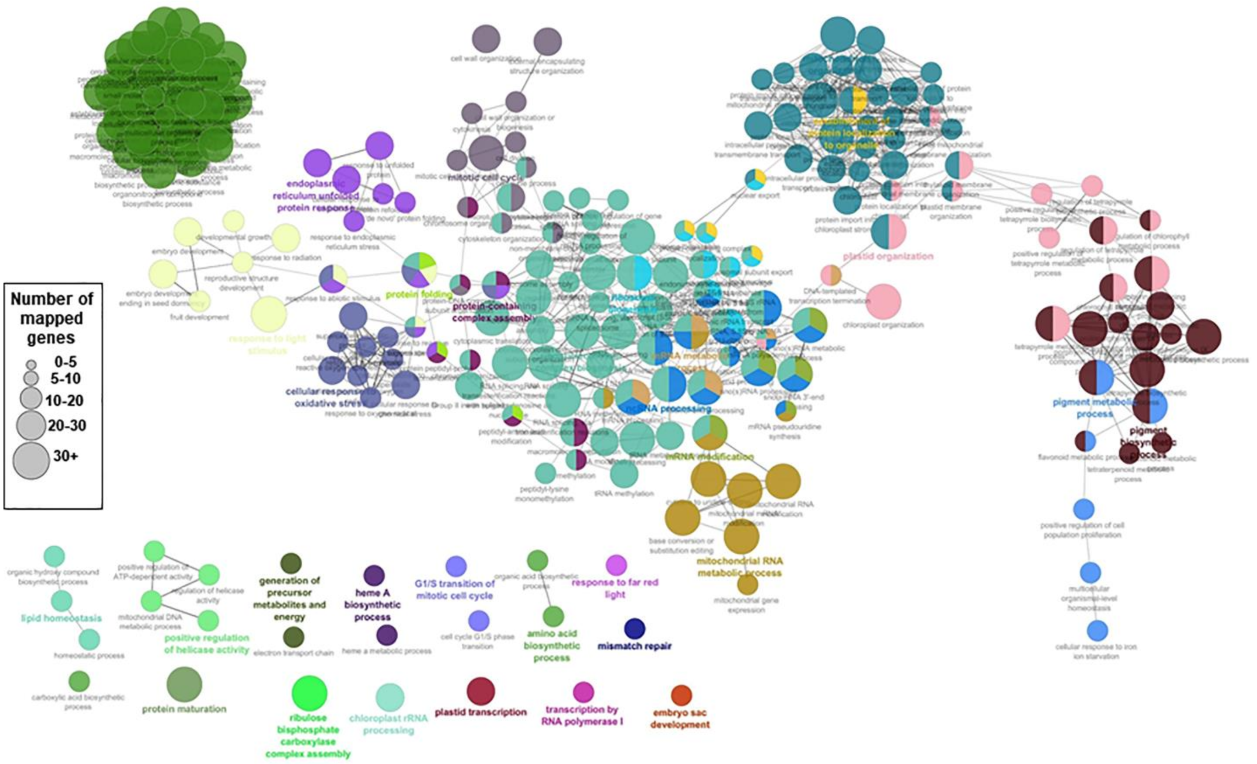

Fig. S11. A network view for the predefined Biological process (BP) GO terms pathways for the WGCNA M11 module. The BP GO terms pathways (P-adjust <0.05) were extracted by g:Profiler website with Benjamini-Hochberg FDR multiple testing correction method. The default ClueGO settings were applied, and the terms are functionally grouped based on shared genes (kappa score). The size of the nodes indicates the number of mapped genes ranged from 0-5, 5-10, 10-20, 20-30, and ≥30 genes. The most significant term defines the name of the group.
